# Revisiting the knowledge concept of mental health literacy and its relationships with stigma

**DOI:** 10.1038/s41598-026-50222-w

**Published:** 2026-04-25

**Authors:** Yifeng Wei, Li Sha, Robert McWeeny, Kylie Johnston, Okan Bulut, Bo Cao, Yanbo Zhang, Andrew Baxter, Wendy Carr, Andrew Greenshaw, Peter Silverstone, Stan Kutcher

**Affiliations:** 1 Department of Psychiatry, University of Alberta, Edmonton, Canada; 2 Faculty of Education, University of Alberta, Edmonton, Canada; 3 Faculty of Education, University of British Columbia, Vancouver, Canada; 4 Department of Psychiatry, Dalhousie University, Dalhousie, Canada

**Keywords:** School-based mental health literacy, Mental health knowledge, Psychometrics, Stigma, Health care, Psychology, Psychology

## Abstract

Mental health knowledge is a core component of school-based Mental Health Literacy (MHL) programs, yet it is often operationalized as a broad multifaceted construct that may obscure distinctions among different types of knowledge. This study re-examined the internal structure and measurement properties of the 30-item knowledge scale from the *Mental Health & High School Curriculum Guide* using data from approximately 440 Grade 9 students and 497 Grade 8 students in western Canada. Confirmatory factor analysis (CFA), item response theory (IRT), and regression analyses were conducted. Results indicated that the original scale was not well represented as a single unidimensional construct. Rather than dismissing the measure, theoretical analysis suggested that the items reflect three components: general knowledge, brain knowledge, and disorder-specific knowledge. The general knowledge and brain knowledge subscales demonstrated more coherent psychometric properties and stable measurement across cohorts, time points, and genders, whereas disorder-specific knowledge showed limited support for a unified latent structure. IRT analyses indicated that the general knowledge subscale was most precise at moderate to higher knowledge levels, whereas the brain knowledge subscale was most precise at low to moderate levels. Multiple regression analyses showed that general knowledge was significantly associated with stigma, whereas brain knowledge was not. These findings highlight the importance of distinguishing among different types of mental health knowledge in MHL research and school-based interventions, suggesting that practitioners may select the original scale, the general knowledge scale, or a combination of the general and brain knowledge scales depending on their purposes.

## Introduction

Abundant research has shown that most adolescents with mental health problems or disorders do not receive adequate or timely help and treatment^1–4^, largely due to stigma and the lack of intentions to seek help^5–7^. Lack of mental health knowledge is a fundamental factor that influences help-seeking intentions and behaviours, which is often exacerbated by factors such as stigma against mental illness^8–10^.

This underscores the crucial role of knowledge in reducing adolescents’ stigma toward mental health and promoting help-seeking for treatment and care when needed. Therefore, a thorough conceptualization of knowledge and the development of a valid scale to measure it are essential prerequisites for effectively implementing and evaluating school-based mental health literacy (MHL) interventions, which are crucial for promoting mental health in children and adolescents.

MHL was originally defined as “knowledge and beliefs about mental disorders which aid their recognition, management, or prevention”^11^ (p.182). This definition has since evolved into a multidimensional construct encompassing (a) “knowledge of how to prevent mental disorders”, (b) “recognition of when a disorder is developing”^5^ (p.231), and (c) “an understanding of mental disorders and their treatments”^12^ (p.155)^[,3^. Thus, mental health knowledge can generally be categorized as either a broad understanding of mental health or disorder-specific knowledge of mental disorders, such as depression and anxiety^13,14^. Accordingly, many school-based MHL interventions employ scales that integrate both general and disorder-specific knowledge components^13–15^. However, a recent systematic review^16^ reported insufficient evidence that Mental Health First Aid improved helping behaviors when outcomes were measured using disorder-specific knowledge scales. This supports the idea that the specificity and type of knowledge assessed may influence the association between knowledge and other MHL components^13–17^. These findings invite researchers to further examine how mental health knowledge is conceptually and empirically structured and operationalized, and to deepen understanding of whether and how the specificity of mental health knowledge shapes its associations with other MHL components, such as stigma. We, in the present study, original 30-item knowledge scale that covers six topics: brain functions, basic facts about mental health and mental illness, information about specific mental disorders, consequences of untreated mental illness, and mental illness treatments^7^. CFA was chosen because it evaluates whether the overall factor structure aligns with the theoretical hypothesis^18^. Then we conceptually regrouped the items into three main subscales: *general knowledge*, *brain knowledge*, and *specific knowledge*. Our approach in this regard aligns with ongoing efforts to theoretically reconceptualize and psychometrically re-evaluate MHL components and their interactions^19–21^.

The prevailing approach to scale design reflects the view that MHL comprises multiple interrelated components; mental health knowledge has therefore typically been measured as a multifaceted construct. For example, the 30-item knowledge scale in the *Mental Health & High School Curriculum Guide* (the Guide) was developed as a multidimensional instrument for integration into junior high and high school curricula^7,21^. However, despite its widespread recognition and application, the internal structure of the knowledge scale within the Guide has received limited systematic examination at both the scale and item levels. Although the scale appears multidimensional, its structure warrants further conceptual and psychometric examination to clarify the distinct knowledge domains it may capture, rather than being dismissed entirely. Such re-evaluation allows us to assess whether distinct knowledge domains demonstrate differential associations with other MHL components, such as stigma.

In addition, many school-based MHL studies lack a thorough psychometric evaluation of the tools used to assess intervention effectiveness^13,18–20^. A systematic review^13^ reported that only 17 of 131 studies included psychometric analyses of the measurement tools, including those assessing knowledge. Consistent with these findings, both Simkiss et al.^14^ and Tullius et al.^17,]^ reported poor internal consistency of the knowledge scale among adolescents. In this context, the present study provides a comprehensive psychometric evaluation of the widely used mental health knowledge scale included in the Guide, a tool widely employed in school-based MHL interventions in many countries. However, despite its widespread use, the psychometric properties of the scale, its component structure representing different knowledge domains, and their relationships with stigma remain insufficiently examined.

Therefore, the current study addressed the following three questions:

1. Is the original 30-item knowledge scale better represented by a unidimensional or multidimensional model based on psychometric analyses (e.g., CFA and IRT)?
2. Do the theoretically derived subscales demonstrate adequate psychometric fit to the data?
3. Are the subscales differentially associated with stigma, thereby supporting their concurrent validity?

## Methods

### Participants

This study included Grade 8 and Grade 9 students drawn from two longitudinal studies examining the impact of the MHL intervention, *The Guide*, conducted in two Canadian school districts. Of the 440 students who completed the pre-test, 240 also completed the post-test from Richmond, British Columbia. British Columbia^21,22^. The Richmond school district has a highly culturally and ethnically diverse environment and hosts many students with Asian heritage, mainly with a Chinese background (43.8% male, 56.2% female). The sample comprised 497 valid Grade 8 participants from Edmonton, Alberta. The Edmonton school district is the second-largest Catholic school division in Canada. Edmonton students (52.5% male, 40.5% female, 7.1% others) participated in the Guide project in Grade 8.

### Measures

The original mental health knowledge was measured with a 30-item questionnaire based on the Guide content. It required participants to choose from one of the three options: ‘true,’ ‘false,’ or ‘do not know^7,23^. Each correct answer was awarded one point, resulting in a maximum mean score of 1, based on how the knowledge variable was computed in the current study. A response of “Do not know” was scored as 0; this option was included to discourage guessing and allow respondents to indicate uncertainty. The knowledge scale has been used to quantify a single latent variable capturing the knowledge component within the MHL framework^12^.

Participants were asked to endorse their responses to 12 items that addressed stigma, ranging from “strongly disagree” to “strongly agree,” using a 7-point Likert scale^23^. Responses were scored between 1 and 7 for each item, with higher scores indicating more positive attitudes, implying lower stigma. The stigma measure yielded robust psychometric properties, with astable Cronbach’s alpha over time (0.84 in both pre- and post-tests)^21^.

### Psychometric analysis

First, univariate outliers, defined as cases with absolute z-scores greater than 3.08, were detected and adjusted in either the predictors or the outcomes^24^. A few multivariate outliers were identified and removed at the 0.001 significance level based on Mahalanobis distance prior to the multiple regression analysis.

An instrument designed to measure a single latent trait should demonstrate unidimensionality—that is, the degree to which all items reflect a common underlying construct^25^. Drawing on this principle, our analysis proceeded in two steps. First, confirmatory factor analysis (CFA) was conducted to evaluate the unidimensionality and homogeneity of the original 30-item knowledge scale^7^(denoted as Original_K). Second, drawing on theoretical distinctions in the literature, the regrouping of the original 30-item scale into three subscales—general, brain-related, and disorder-specific knowledge—was guided by the content of the Guide intervention, which addresses these three aspects of mental health knowledge. The content of the 30 items was further validated by mental health professionals, researchers, and members of the youth advisory group who collaborated with the research team. In the analyses that follow, these subscales are labeled General_K, Brain_K, and Specific_K, respectively; the classification was theoretically guided and informed by a content analysis of the 30 items. Specifically, the general knowledge (14 items) reflects a psychosocial and broad understanding of mental health and illness (e.g., “Feelings are controlled mostly by the heart.”). the brain-related knowledge (6 items) focuses on the biological and neurological mechanisms underlying body–mind interactions in relation to mental health (e.g., “Mental health and mental illness both involve the brain and how it functions”). The disorder-specific knowledge refers to one’s understanding of specific mental disorders (e.g., depression) and was assessed using the 10 items (e.g., “Panic attacks in Panic Disorder happen as a result of stresses in the environment”).

Based on this theory-based categorization, confirmatory factor analysis (CFA) was conducted to evaluate the unidimensionality of each scale and to examine measurement invariance across time (pre- vs. post-test), gender (male vs. female), and cohorts (Richmond vs. Edmonton). Model fit was considered acceptable if the following criteria were met: χ²/df < 4.00, CFI ≥ 0.90, RMSEA < 0.08, and standardized factor loadings ≥ 0.30. As shown in Tables 1 and 2, Both the general knowledge and brain knowledge subscales demonstrated acceptable model fit and were retained for subsequent analyses. Whereas the disorder-specific knowledge subscale did not meet the criterion for adequate unidimensionality across cohorts or over time and was therefore excluded from subsequent multiple regression analyses examining its association with stigma. Multi-group CFAs (see Table 2) was conducted to examine measurement invariance across cohorts and time points (Richmond vs. Edmonton; pre-test vs. post-test), as well as across cohorts and gender (Richmond vs. Edmonton; male vs. female). These analyses assessed whether the factor structure of the subscales remained stable across samples, intervention stages, and gender groups.

Item Response Theory (IRT) analyses were conducted to estimate item parameters and evaluate overall scale performance^26^. First, to determine the appropriate IRT model (1PL vs. 2PL), model comparisons were conducted for each scale within each cohort using the Akaike Information Criterion (AIC), the Bayesian Information Criterion (BIC), and the sample-size adjusted Bayesian Information Criterion (SABIC) (see Table 2). Lower values of these indices indicate better model fit and greater model parsimony. In addition, chi-square difference tests were conducted to assess whether the 2PL model provided a significantly better fit than the 1PL model. Based on these comparisons, the present study focused on two item parameters: discrimination and difficulty. Item discrimination indicates how well an item differentiates among individuals with different levels of the latent trait (θ), whereas item difficulty represents the trait level associated with a 50% probability of a correct response.

**Fig. 1:**
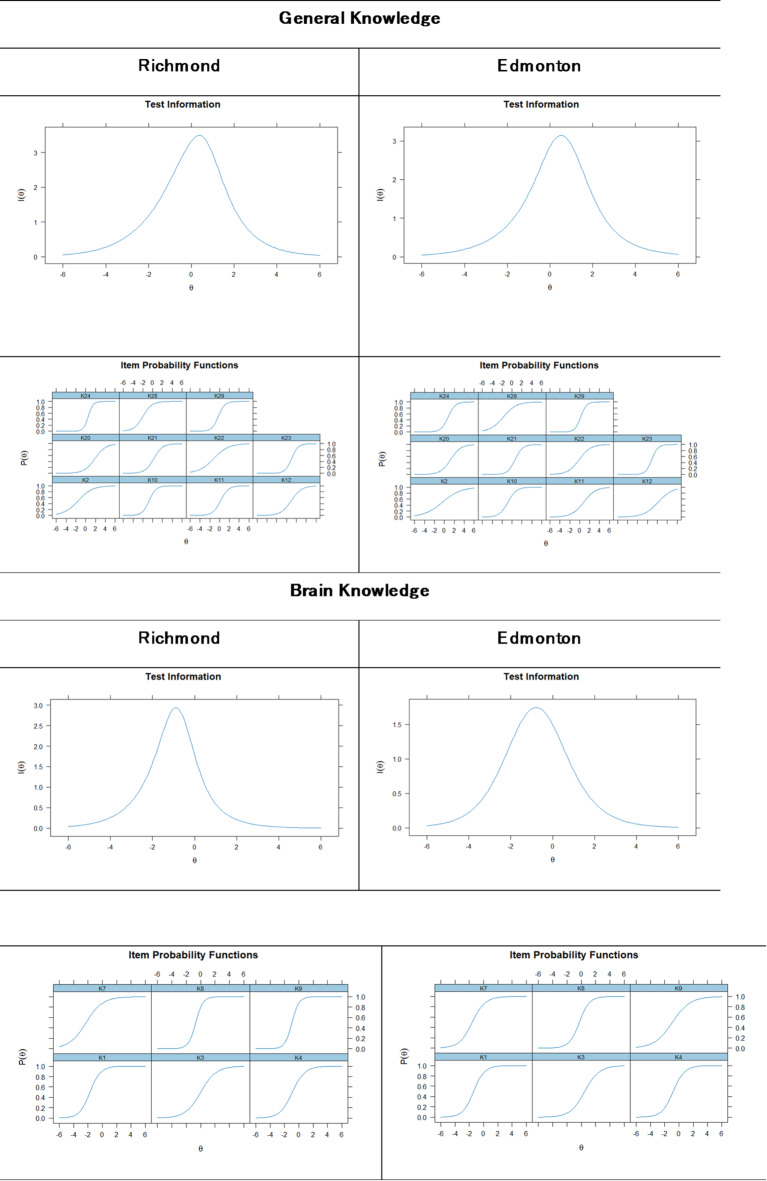
Test Information and Item Probability Functions of the Two Knowledge Subscales for Both Cohorts.

The overall performance of the scales was further evaluated using Test Information Functions (TIFs) and Item Probability Functions (IPFs; also known as Item Characteristic Curves, ICCs) (Fig. 1). TIFs reflect measurement precision across levels of θ, whereas IPFs illustrate the probability of a correct response as a function of θ, with steeper slopes indicating greater item discrimination.

Concurrent validity refers to the extent to which a measure correlates with other variables that are theoretically or empirically expected to be related when assessed at the same time point. In the present study, concurrent validity was evaluated by examining the associations between the knowledge subscales and stigma using correlation and regression analyses. Prior research has shown relatively stable associations between knowledge and stigma^12,19^. This approach allows for a more nuanced assessment of whether general or brain-related knowledge is more strongly associated with stigma.

Finally, the sample sizes in both cohorts (*N* ≈ 440–523) exceeded commonly recommended thresholds for CFA and 2PL IRT modeling^27^, as well as for multiple regression analyses. All CFA and IRT analyses were conducted in R^28^. CFA models were estimated using the WLSMV (Weighted Least Squares Mean and Variance adjusted) estimator, which is appropriate for binary items (0/1), whereas IRT models were estimated using marginal maximum likelihood (MML)^29,30^. SPSS 29.0 was used to conduct correlational and multiple regression analyses.

## Results

### Knowledge scales: CFA and IRT analyses

The 30-item knowledge scale did not reach commonly recommended thresholds for an acceptable single-factor model (CFI/TLI ≥ 0.90) across both cohorts (Richmond vs. Edmonton) and time points (pre-test vs. post-test) (see Table 1). Accordingly, the scale was not treated as a single latent factor in subsequent analyses (e.g., IRT and regression). In contrast, both General_K and Brain_K demonstrated good model fit, whereas Specific_K showed poor fit, with CFI values ranging from 0.52 to 0.81. To achieve acceptable model fit, three items (i.e., Items 6, 25, and 30) with factor loadings below 0.30 were removed from the 14-item General_K subscale. Notably, the original scale showed moderate but suboptimal model fit, suggesting that a single-factor structure may not fully capture its internal structure, whereas the Specific_K subscale demonstrated substantially poorer model fit, indicating that this subscale may not be adequately represented by a single factor.

Meanwhile, multi-group CFA analyses (see Table 2) supported stable measurement invariance for the four scales. Configural, metric, and scalar invariance were supported under both grouping schemes—cohort and time point (Richmond vs. Edmonton; pre-test vs. post-test) and cohort and gender (Richmond vs. Edmonton; male vs. female)—with minimal changes in model fit indices (ΔCFI ≤ 0.01; ΔRMSEA ≤ 0.01). Across varying contexts, these results further support the unidimensional structure of the General_K and Brain_K subscales, while offering more limited support for the unidimensional structure of the original scale and the Specific_K subscale. As a result, the latter two were excluded from subsequent IRT and multiple regression analyses.

**Table 1:** The model-fit parameters across cohorts and between genders.

	Richmond	Edmonton
Pre-test		
Original_K	χ^2^(405) = 706.14, *p <* .001, CFI=0.83, TLI=0.82, RMSEA=0.04	χ^2^(435) = 2831.43, *p <* .001, CFI=0.81, TLI=0.80, RMSEA=0.05
General_K	χ^2^(55) = 817.82, *p*<.001, CFI=0.93, TLI=0.91, RMSEA=0.05	χ^2^(55) = 788.21, *p* < .001, CFI=0.96, TLI=0.95, RMSEA=0.04
Brain_K	χ^2^(9) = 5.96, *p =* .70, CFI = 1.00, TLI = 1.02, RMSEA<0.001	χ^2^(15) = 242.88, *p <* .001, CFI = 1.00, TLI = 1.02, RMSEA<0.001
Specific_K	χ^2^(45) = 215.31, *p*<.001, CFI=0.81, TLI=0.76, RMSEA=0.05	χ^2^(45) = 259.96, *p <* .001, CFI=0.52, TLI=0.38, RMSEA=0.08
Post-test		
Original_K	χ^2^(405) = 812.58, *p <* .001, CFI=0.71, TLI=0.68, RMSEA=0.06	χ^2^(435) = 2426.09, *df =* 405, *p <* .001, CFI=0.80, TLI=0.78, RMSEA=0.05
General_K	χ^2^(55) = 649.98, *p*<.001, CFI=0.93, TLI=0.91, RMSEA=0.05	χ^2^(55) = 838.08, *p* < .001, CFI=0.97, TLI=0.96, RMSEA=0.03
Brain_K	χ^2^(9) = 3.98, *p =* .90, CFI = 1.00, TLI = 1.84, RMSEA<0.001	χ^2^(15) = 178.01, *p <* .001, CFI=0.90, TLI=0.83, RMSEA=0.07
Specific_K	χ^2^(45) = 268.52, *p*<.001, CFI=0.54, TLI=0.41, RMSEA=0.09	χ^2^(45) = 219.94, *p <* .001, CFI=0.55, TLI=0.42, RMSEA=0.07
	Male	Female
Richmond (Pre-test)		
General_K	χ^2^(55) = 316.38, *p*<.001, CFI=0.88, TLI=0.85, RMSEA=0.06	χ^2^(55) = 513.61, *p*<.001, CFI=0.94, TLI=0.92, RMSEA=0.05
Brain_K	χ^2^(15) = 144.61, *p*<.001, CFI=0.96, TLI=0.93, RMSEA=0.06	χ^2^(15) = 170.03, *p*<.001, CFI = 1.00, TLI = 1.03, RMSEA=0.05
Edmonton (Pre-test)		
General_K	χ^2^(55) = 395.04, *p*<.001, CFI=0.96, TLI=0.95, RMSEA=0.03	χ^2^(55) = 374.93, *p*<.001, CFI=0.97, TLI=0.96, RMSEA=0.03
Brain_K	χ^2^(15) = 206.03, *p*<.001, CFI=0.99, TLI=0.99, RMSEA=0.02	χ^2^(15) = 54.54, *p*<.001, CFI = 1.00, TLI = 1.09, RMSEA<0.001

**Table 2:** Measurement invariance of the various scales across cohorts, time points, and gender.

	Model	CFI	TLI	RMSEA	ΔCFI	ΔTLI	ΔRMSEA
Cohort and Time							
Original Knowledge	Configural	0.80	0.78	0.05	NA	NA	NA
	Metric	0.81	0.81	0.05	0.01	0.03	−0.003
	Scalar	0.81	0.81	0.05	0.00	0.00	0.00
General Knowledge	Configural	0.95	0.94	0.05	NA	NA	NA
	Metric	0.95	0.95	0.04	0.00	0.01	0.00
	Scalar	0.95	0.95	0.04	0.00	0.00	0.00
Brain Knowledge	Configural	0.99	0.98	0.03	NA	NA	NA
	Metric	0.95	0.94	0.05	− 0.04	− 0.04	0.02
	Scalar	0.95	0.94	0.05	0.00	0.00	0.00
Specific knowledge	Configural	0.61	0.50	0.07	NA	NA	NA
	Metric	0.55	0.52	0.07	− 0.06	0.02	0.00
	Scalar	0.55	0.52	0.07	0.00	0.00	0.00
Cohort and Gender							
General Knowledge	Configural	0.94	0.93	0.05	NA	NA	NA
	Metric	0.93	0.92	0.05	− 0.01	0.00	0.00
	Scalar	0.93	0.92	0.05	0.00	0.00	0.00
Brain Knowledge	Configural	1.00	1.01	0.00	NA	NA	NA
	Metric	1.00	1.00	0.01	− 0.01	− 0.01	0.01
	Scalar	1.00	1.00	0.01	0.00	0.00	0.00

Note that “NA” reflects the absence of change indices for the baseline (configural) model.

The IRT analyses produced four sets of results, all based on the pre-test data from both cohorts, as the pre-test sample provided the largest and most suitable sample size in the Richmond cohort compared with the smaller post-test sample. First, the 2PL model was selected over the 1PL model based on the model comparisons in Table 3 (Part A). The information criteria (AIC, BIC, and SABIC) favored the 2PL model across cohorts for both scales, except for the brain knowledge scale in the Edmonton cohort. Significant Δχ² tests (*p* < .05) further supported the 2PL model. Moreover, the 2PL model was retained to estimate item discrimination and difficulty parameters at both the scale and item levels. Second, as shown in Table 3 (Part B), the M2 statistics indicated that both unidimensional scales demonstrated acceptable to excellent fit across cohorts (CFI = 0.93–1.00), consistent with the CFA results in Table 1.

**Table 3:** Comparison of 1PL and 2PL Models and Fit of the Selected 2PL Models Across Scales and Cohorts.

Scale	Cohort	Model	AIC	SABIC	BIC	Δχ²(df)	*p*
Part A Model Comparison Between 1PL and 2PL							
Brain Knowledge	Richmond	1PL	2710.72	2717.09	2739.31		
		2PL	2703.16	2714.09	2752.18	17.55(5)	0.004
	Edmonton	1PL	3458.22	3465.47	3487.68		
		2PL	3462.58	3474.99	3513.08	5.64 (5)	0.34
General Knowledge	Richmond	1PL	5354.54	5365.47	5403.55		
		2PL	5337.31	5357.35	5427.17	37.23 (10)	< 0.001
	Edmonton	1PL	6422.24	6434.65	6472.74		
		2PL	6394.32	6417.08	6486.91	47.92 (10)	< 0.001

Scale	Cohort	M2 (df)	*p*	RMSEA	95% CI	SRMSR	CFI	TLI
Part B. Model Fit Indices for the Selected 2PL Models								
General Knowledge	Richmond	95.80 (44)	< 0.001	0.05	[0.04, 0.07]	0.05	0.93	0.92
	Edmonton	70.46 (44)	0.01	0.03	[0.02, 0.05]	0.04	0.97	0.96
Brain Knowledge	Richmond	4.89 (9)	0.84	0.00	[0.00, 0.03]	0.02	1.00	1.02
	Edmonton	6.08 (9)	0.73	0.00	[0.00, 0.04]	0.02		

Third, the total test information curves for the general and brain knowledge scales (Fig. 1) were unimodal and approximately symmetric, providing further support for their unidimensional structure across the Richmond and Edmonton cohorts. For the general knowledge scale, peak information exceeded 3.0 in both cohorts and occurred around average to slightly above-average trait levels (θ ≈ 0–1), revealing the greatest measurement precision for individuals with moderate levels of general knowledge. In contrast, the brain knowledge scale showed peak information at slightly below-average to average trait levels (θ ≈ −1 to 0), suggesting that its items were relatively easier and most informative for individuals with lower-to-moderate levels of brain knowledge. Peak information for brain knowledge was also somewhat lower overall, particularly in the Edmonton cohort, showing reduced measurement precision relative to the general knowledge scale. For both scales, information declined toward the lower and upper extremes of the trait continuum (θ < −3 and θ > +3), indicating reduced precision for individuals with very low or very high levels of knowledge. Despite minor differences in peak magnitude and location, the overall shapes of the information curves were broadly similar across the Richmond and Edmonton cohorts, reflecting stable measurement characteristics across samples.

Item-level parameters from the 2PL model (see Table 4; Fig. 1) showed that most items on the general and brain knowledge scales exhibited moderate to high discrimination. For the general knowledge scale, discrimination ranged from 0.66 to 2.05 in the Richmond cohort and from 0.53 to 1.70 in Edmonton, with difficulty parameters largely centered around the middle of the latent trait continuum. For the brain knowledge scale, discrimination ranged from 0.86 to 2.04 in Richmond and from 0.80 to 1.39 in Edmonton, while difficulty parameters were predominantly negative, demonstrating that these items were relatively easier for participants. Overall, discrimination and difficulty parameters showed broadly comparable patterns across cohorts. The item probability curves in Fig. 1 provide complementary visual illustrations; for example, Item 24 in the general knowledge scale shows a particularly steep slope, reflecting its high discrimination in both cohorts.

**Table 4:** Item Discrimination and Difficulty Values of The Scales Across Cohorts (Disc: discrimination, Diff: difficulty).

General Knowledge	Richmond		Edmonton	
	Disc	Diff	Disc	Diff
2. People who have mental illness can at the same time have mental health.	0.69	-1.51	0.53	− 0.35
10.Feelings are controlled mostly by the heart	1.4	− 0.70	1.15	− 0.81
11.Most people who have a mental illness don’t get well and stay well with treatment.	1.24	0.22	0.83	0.81
12.Vitamins and meditation are good treatments for most mental illnesses.	0.94	0.99	0.69	2.18
20. Suicide in young people is mostly the result of the stress of being a teenager.	0.9	1.93	0.98	1.28
21.Self-harming behaviors are the same as suicide attempts	1.12	0.26	1.34	0.64
22.Treatment of mental disorders has three purposes including relieving symptoms, restoring functioning, and promoting recovery	0.66	-1.17	0.88	− 0.11
23.Mental illnesses are caused by usual stresses of everyday life	1.48	1.05	1.58	0.88
24.All mental distress will develop into mental illness overtime	2.05	0.54	1.39	0.78
28.Good social relationships and exercise BOTH help to promote good mental health	1.09	-1.98	0.76	1.74
29.Occasional sadness and anger are signs of poor mental health	1.54	-0.24	1.7	0.21
	Richmond		Edmonton	
Brain Knowledge	Disc	Diff	Disc	Diff
1.Mental health and mental illness both involve the brain and how it functions	1.34	-1.71	1.3	-1.38
3.The brain can affect the way the body functions but the body can not affect the way the brain functions	0.93	0.02	0.98	0.51
4.Different brain functions are all controlled by various neuron circuits forming networks communicating with each other	1.15	− 0.89	1.24	− 0.78
7.The brain acts to help control the functioning of the heart, lungs, and fingers	0.86	-2.27	1.09	-1.58
8.Both genetic problems and infections can cause the brain to get sick and stop functioning normally	1.96	− 0.68	1.39	− 0.27
9.Cognition, body movements and signaling are ALL functions controlled by the brain	2.04	− 0.94	0.80	− 0.91

### Concurrent validity of the knowledge scales: relationships with stigma

In addition to examining the internal psychometric properties of the scales, the concurrent validity of the two identified knowledge constructs was assessed through their relationships with stigma. Table 5 shows that that variables can be considered nearly normally distributed^24^. The Cronbach’s alpha for General_K and Brain_K demonstrated marginally acceptable internal consistency (0.61 < α < 0.70)^31,32^. The zero-order correlations between General_K and Brain_K were moderate in both cohorts (*r* = .48 in Richmond; *r* = .44 in Edmonton). In both cohorts, stigma was more strongly correlated with general knowledge than with brain knowledge, a pattern further supported by multiple regression analysis (see Table 5).

**Table 5:** Descriptive Statistics and Pearson correlations Across Two Cohorts.

Descriptive Statistics (Pre-test)								
	*N*	Min	Max	Mean	STD	Skewness	Kurtosis	Alpha
Richmond								
General_K	439	0	1	0.50	0.23	0.12	− 0.49	0.70
Brain_K	439	0	1	0.73	0.25	− 0.94	0.38	0.61
Stigma	434	2.5	7	5.46	0.89	− 0.37	− 0.62	0.85
Edmonton								
General_K	497	0	1	0.43	0.23	0.21	− 0.68	0.68
Brain_K	497	0	1	0.65	0.26	− 0.51	− 0.43	0.63
Stigma	492	2.25	7	5.38	0.93	− 0.46	− 0.28	0.84
Pearson Correlations (Pre-test)								
	General_K				Brain_K			
Richmond								
General_K	1.00							
Brain_K	0.48**				1.00			
Stigma	0.37**				0.24**			
Edmonton								
General_K	1.00							
Brain_K	0.44**				1.00			
Stigma	0.34**				0.16**			

*p<.05, **p<.01.

The multiple regression models (see Table 6) were significant for both cohorts. general knowledge significantly predicted the outcome in both cohorts (β = 0.33, *p* < .001), whereas brain knowledge was not a significant predictor. Notably, higher stigma scores in this study indicate more positive attitudes (i.e., lower stigma); therefore, the regression results showed a negative association between adolescents’ general knowledge and stigmatizing attitudes toward mental health issues. Variance inflation factors were low (VIFs = 1.24–1.30), indicating no concerns regarding multicollinearity. An a priori power analysis was conducted using G*Power 3.1.9.7 for the F-test in multiple regression with two predictors. Assuming α = 0.05, power (1 − β) = 0.95, and a small-to-moderate effect size (f² = 0.05), the required minimum sample size was 312 participants. The available samples in the Richmond (*N* = 433) and Edmonton (*N* = 491) cohorts exceeded this requirement for the two-predictor regression models. The regression models explained a meaningful proportion of variance (Richmond: Adjusted *R*^2^=0.14; Edmonton: Adjusted *R*^2^=0.11), corresponding to small-to-moderate effect sizes (Richmond: f^2^=0.16; Edmonton: f^2^=0.12). Therefore, although the models explained a relatively small proportion of variance (Adjusted R² = 0.14 and 0.11), the regression analyses were adequately powered.

**Table 6:** Summary of Multiple Linear Regression for Stigma in Both Cohorts (Pre-test data only).

Predictor	B	SE B	β	t	*p*	95% CI for B	VIF
Richmond (F(2,431) = 35.09, *p* < .001, Adjusted *R*^*2*^=0.14)							
General_K	1.28	0.20	0.33	6.47	< 0.001	[0.89, 1.67]	1.30
Brain_K	0.28	0.18	0.08	1.54	0.13	[-0.08, 0.64]	1.30
Edmonton (*F*(2,489) = 31.62, *p* < .001, Adjusted *R*^*2*^=0.11)							
General_K	1.37	0.20	0.33	6.96	< 0.001	[0.99, 1.76]	1.24
Brain_K	0.07	0.17	0.02	0.39	0.69	[-0.27, 0.41]	1.24

## Discussion

It is important to emphasize that the present study does not seek to challenge the theoretical conceptualization or the multidimensional nature of mental health knowledge widely recognized in the literature. Rather, it re-examines the internal structure of the widely used 30-item knowledge scale within the Guide^7,23^ to explore whether it can be represented as distinguishable components, such as general knowledge, brain-related knowledge, and disorder-specific knowledge.

First, CFA results supported unidimensional structures for the general knowledge and brain knowledge scales across cohorts, time points, and genders. The IRT analyses yielded consistent evidence, with acceptable to excellent model fit based on the M2 statistic and related indices. However, their marginally acceptable Cronbach’s alpha values (between 0.61 and 0.70; see Table 5) represent a limitation of the present study. The convergence of findings across SEM- and IRT-based approaches is noteworthy, as these frameworks rely on different parameterizations and estimation principles. Both approaches supported the same dimensional structure, strengthening the robustness of the scales. Moreover, the IRT analyses extend the CFA findings by providing item-level information on measurement precision, discrimination, and difficulty, offering additional insights into the psychometric properties of the scales. To our knowledge, this study is the first to integrate both methods to evaluate a mental health knowledge scale in school-based MHL interventions.

Although the incremental fit indices (CFI and TLI) provided limited support for a strictly unidimensional structure of the original 30-item scale across cohorts and time points, the scale may still function as a multifaceted measure in some contexts, such as when a broad composite indicator of knowledge is sufficient and strict model fit is not required. Rather than rejecting the original scale, our analyses suggest that mental health knowledge can also be assessed using separate unidimensional scales representing distinct knowledge domains. This approach allows researchers and practitioners to use the scales in a more targeted manner. In practice, users may select individual subscales or combine them depending on their analytic goals. This approach may therefore provide a more informative framework for understanding how different components of mental health knowledge develop and interact in school-based MHL interventions.

Moreover, the IRT analyses provide additional insights into the structural features of adolescents’ mental health knowledge across cohorts (see Fig. 1; Table 4). Most items demonstrated moderate to high discrimination, indicating that both the general knowledge and brain knowledge items effectively differentiated students with varying levels of these two forms of mental health knowledge.

The difficulty parameters revealed clear differences between the two knowledge domains. The general knowledge items showed a wider range of difficulty levels. A few items required relatively higher levels of knowledge to answer correctly, particularly those addressing misconceptions about the causes of mental illness and suicide. For example, Item 20 had the highest difficulty parameter (see Table 4, “Suicide in young people is mostly the result of the stress of being a teenager.”). Whereas, brain knowledge items were generally easier, with most difficulty parameters falling below zero across both cohorts. This pattern may reflect students’ existing understanding of basic brain-related concepts, such as the brain’s role in regulating bodily functions and cognition. For example, the item with the lowest difficulty parameter was “The brain acts to help control the functioning of the heart, lungs, and fingers” (see Table 4). This finding suggests that brain-related concepts may serve as an accessible entry point for school-based MHL interventions. By comparison, general knowledge items covering topics such as mental illness, treatment, and suicide tended to exhibit higher difficulty parameters, suggesting that adolescents may require greater support in developing understanding in these areas. These findings suggest that further research is needed to examine how different domains of mental health knowledge interact and the mechanisms through which they influence stigma, help-seeking intentions, and other MHL components.

The multiple regression analyses (Table 6) showed that general knowledge significantly predicted stigma in both cohorts; however, the explained variance was modest (adjusted R² between.11 and 0.14), indicating that knowledge accounts for only a limited portion of variability in adolescents’ stigmatizing attitudes, which represents a limitation of the present study. This finding is consistent with previous research suggesting that stigma is shaped by multiple psychological and social influences beyond knowledge alone, including the type of mental illness, treatment engagement, socioeconomic status (SES), gender, and cultural context^33^.

Many general knowledge items address misconceptions about the causes, treatability, and consequences of mental illness, which may be conceptually linked to key dimensions of stigma such as blame, perceived incompetence, and social distancing. For example, recognizing that many people with mental illness can recover with appropriate treatment (Item 11: “Most people who have a mental illness don’t get well and stay well with treatment”) may challenge stereotypes that individuals with mental illness are incapable of working (Stigma Item 1: “Most people with a mental illness are too disabled to work”).

Notably, given the large sample sizes and adequate statistical power indicated by the G*Power analysis, the non-significant effect of brain knowledge on stigma is unlikely to be attributable to insufficient power. Taken together, these findings suggest that the relationship between mental health knowledge and other MHL components may vary depending on the type and specificity of the knowledge assessed, consistent with previous studies^7,10,14,15,17^.

One possible explanation for the non-significant association between brain knowledge and stigma is that brain knowledge items were primarily designed to enhance understanding of the biological basis of mental health rather than to directly address stigma-related attitudes. Among the six brain knowledge items, only Item 1 (“Mental health and mental illness both involve the brain and how it functions”) explicitly refers to mental health, whereas the remaining items focus on general brain functions without direct reference to mental health or mental illness. This lack of explicit mental health framing may help explain why brain knowledge was not associated with stigma in either cohort. Moreover, prior research suggests that biogenetic explanations of mental illness do not necessarily reduce stigma and may have mixed effects on stigma-related attitudes^34,35^. Therefore, the absence of a significant association between brain-related knowledge (e.g., Item 8: “Both genetic problems and infections can cause the brain to get sick and stop functioning normally.”) and stigma in the present study may reflect the complex and sometimes indirect relationship between biological knowledge and stigma.

Finally, the 10-item disorder-specific knowledge subscale exhibited poor unidimensionality and limited internal homogeneity in both cohorts (see Tables 1 and 2). This limitation undermines the psychometric basis for treating the items as a reliable single latent construct. Consequently, this complicates attempts to examine how this form of knowledge relates to other components of MHL when analyzed as a single scale. Nevertheless, as an integral component of mental health knowledge, disorder-specific knowledge remains important to investigate further in order to better understand its potential role in shaping adolescents’ stigma and help-seeking attitudes and behaviors. Accordingly, the absence of such analyses represents a limitation of the present study and should be addressed in future research.

Individuals’ knowledge about specific mental disorders often varies substantially across conditions—for example, recognition rates tend to differ across disorders such as depression and schizophrenia—suggesting that disorder-specific knowledge may not necessarily form a single coherent knowledge domain^36^.^37^(Jorm et al., 1997; Shahwan et al., 2026). Therefore, it may be unrealistic to expect these items to form a single unifying construct that demonstrates acceptable model fit and internal consistency (e.g., Cronbach’s alpha) under strict psychometric criteria. Accordingly, one of our follow-up studies will employ IRT methods to examin**e** this form of knowledge at the item level, by evaluating each item’s discrimination and difficulty parameters. This approach is expected to provide detailed item-level psychometric information and help identify items with extreme or atypical difficulty parameters that may partly explain the scale’s poor overall structure if a unidimensional disorder-specific knowledge scale is pursued. For example, our initial analysis showed that Item 19 (see Appendix) had an extremely high difficulty parameter (19.71). However, given the psychometric challenges of forming a reliable single construct, it may still be appropriate to examine the relationships between disorder-specific knowledge and other components of MHL at the item level rather than the scale level. Such an approach would allow us to identify which types of mental disorder knowledge (e.g., depression or anxiety) are most strongly associated with stigma and help-seeking intentions. These findings could provide useful insights for designing more tailored and effective school-based MHL interventions and for deepening our understanding of the complex structure of mental health knowledge and its nuanced associations with stigma and help-seeking.

## Conclusions

First, the findings suggest that the original 30-item mental health knowledge scale within the Guide may not be adequately represented as a single unidimensional construct, indicating potential heterogeneity in the knowledge domains captured by its items. Second, separating the items into general and brain-related knowledge components produced more coherent psychometric properties and more stable measurement across cohorts and time points, as demonstrated by both CFA and IRT analyses. Third, the two knowledge components showed different relationships with stigma: general knowledge was significantly associated with stigma, whereas brain-related knowledge was not. Taken together, these results highlight the importance of distinguishing among different types of mental health knowledge in MHL research and school-based interventions, suggesting that practitioners may select the original scale, the general knowledge scale, or a combination of the general and brain knowledge scales depending on their purposes.

## Data Availability

The data that support the findings of this study are available from the corresponding author upon reasonable request.

## Appendix: The Mental Health Knowledge Scale

**Table:**

General Knowledge
2.People who have mental illness can at the same time have mental health.
6.Every person’s mood can fluctuate up and down naturally.
10.Feelings are controlled mostly by the heart.
11.Most people who have a mental illness don’t get well and stay well with treatment.
12.Vitamins and meditation are good treatments for most mental illnesses.
20.Suicide in young people is mostly the result of the stress of being a teenager.
21.Self-harming behaviors are the same as suicide attempts.
22.Treatment of mental disorders has three purposes including, relieving symptoms, restoring functioning, and promoting recovery.
23.Mental illnesses are caused by usual stresses of everyday life.
24.All mental distress will develop into mental illness overtime.
25.Mental health can be improved by leading a physically healthy life.
28.Good social relationships and exercise BOTH help to promote good mental health.
29.Occasional sadness and anger are signs of poor mental health.
30.The phenomenon of craving drives substance abuse.
Brain-related Knowledge
1.Mental health and mental illness both involve the brain and how it functions.
3.The brain can affect the way the body functions but the body can not affect the way the brain functions.
4.Different brain functions are all controlled by various neuron circuits forming networks communicating with each other.
7.The brain acts to help control the functioning of the heart, lungs, and fingers.
8.Both genetic problems and infections can cause the brain to get sick and stop functioning normally.
9.Cognition, body movements and signaling are ALL functions controlled by the brain.
Disorder-Specific Knowledge
5.Most people who experience traumatic events such as a car accident will develop a post traumatic stress disorder.
13.People who have schizophrenia often get a split personality.
14.Depression and Bipolar Disorder are two examples of the type of mental illnesses called mood disorders.
15.An anxiety disorder happens when a person’s brain detects the presence of danger – such as a dog attacking.
16.Panic attacks in Panic Disorder happen as a result of stresses in the environment.
17.People with social anxiety disorder often feel as if they are being scrutinized and judged by others.
18.An SSRI medicine and cognitive behavioral therapy are given together to effectively treat Obsessive Compulsive Disorder.
19.Attention Deficit Hyperactivity Disorder has three components including attention problems, hyperactivity, and anxiety.
26.If a person feels sad for a few days in a row, they likely have a Depression.
27.Young people with Bulimia Nervosa often starve themselves and exercise excessively.

© Stan Kutcher, 2016.
